# WestREN: a description of an Irish academic general practice research network

**DOI:** 10.1186/1471-2296-11-74

**Published:** 2010-10-06

**Authors:** Kim E Kavanagh, Niamh O'Brien, Liam G Glynn, Akke Vellinga, Andrew W Murphy

**Affiliations:** 1 Discipline of General Practice, NUI Galway, 1 Distillery Road, Newcastle, Galway, Ireland; 2 Irish College of General Practitioners, 4/5 Lincoln Place, Dublin 2, Ireland

## Abstract

**Background:**

Primary care research networks have been established internationally since the 1960s to enable diverse practitioners to engage in and develop research and education and implement research evidence.

The newly established Western Research and Education Network (WestREN) is one such network consisting of a collaboration between the Discipline of General Practice at NUI Galway and 71 West of Ireland general practices. In September 2009 all member practices were issued with a questionnaire with two objectives: to describe the structure and characteristics of the member practices and to compare the results to the national profile of Irish general practice.

**Methods:**

A postal survey was used followed by one written and one email reminder.

**Results:**

A response rate of 73% (52/71) was achieved after two reminders.

Half of practices were in a rural location, one quarter located in an urban setting and another quarter in a mixed location.

Ninety-four per cent of general practitioners practice from purpose-built or adapted premises with under 6% of practices being attached to the general practitioner's residence. Over 96% of general practitioners use appointment systems with 58% using appointment only.

All practices surveyed were computerised, with 80% describing their practices as 'fully computerised'. Almost 60% of general practitioners are coding chronic diagnoses with 20% coding individual consultations. Twenty-five per cent of general practitioners were single-handed with the majority of practices having at least two general practitioners, and a mean number of general practitioners of 2.4. Ninety-two per cent of practices employed a practice nurse with 30% employing more than one nurse.

Compared to the national profile, WestREN practices appear somewhat larger, and more likely to be purpose-built and in rural areas. National trends apparent between 1982 and 1992, such as increasing computerisation and practice nurse availability, appear to be continuing.

**Conclusions:**

WestREN is a new university-affiliated general practice research network in Ireland. Survey of its initial membership confirms WestREN practices to be broadly representative of the national profile and has provided us with valuable information on the current and changing structure of Irish general practice.

## Background

A network has been described in organisational management literature as a set of nodes and the set of ties representing some relationship between the nodes [1].

In primary care literature, networks have been described as sets of pathways for people and ideas to come together [2]. Networks used for research purposes have been established internationally since the 1960s to enable diverse practitioners to engage in, and develop research and education in primary health care, and implement research evidence [2]. They can produce multidisciplinary coalitions of researchers, provide widespread ownership of research activity and motivate members to disseminate research findings quickly. There is substantial literature which confirms that research networks are feasible and capable of important research that can affect virtually everyone [3]. Internationally influential primary care research infrastructures have been developed in the United Kingdom [4] but to date no such network has existed in Irish primary care.

**Figure 1:**
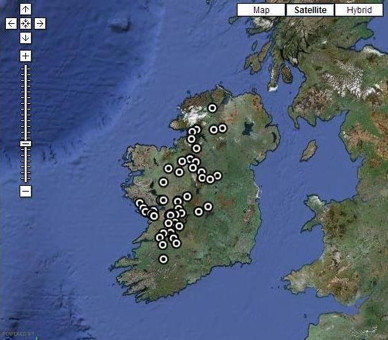
**Location of WestREN practices**. This map demonstrates the geographical locations and distribution of WestREN member practices in Ireland, with each circular black and white symbol representing one practice. Data was correct at time of article submission. An up to date map can be accessed at http://westren.nuigalway.ie, where placing a cursor over a practice symbol will display practice name and contact details.

In October 2009, the Western Research and Education Network (WestREN) was launched, consisting of a collaboration between the Discipline of General Practice, National University of Ireland (NUI) Galway and 71 West of Ireland general practices (see Figure 1). This is an Irish university-affiliated general practice oriented research network, whose defining features are that it is comprised of practices that formally agreed to participate, were openly recruited and cover a wide geographical area. Its mission is to support primary care research and education in order to improve the quality of care delivered to patients in the community in the West of Ireland. This paper describes the participating practices and compares them to the national Irish general practice profile [5].

## Methods

All practices in the NUI Galway undergraduate general practice tutor database were invited in writing to join WestREN in June 2009. Interested parties were asked to provide details of a practice contact person, and computerisation status. In September 2009 a membership certificate and questionnaire were sent to all member practices. This questionnaire (attached as Additional File 1) was devised based on the original questionnaire used by O'Dowd et al. [5] in their national surveys of Irish general practitioners in 1992 and 2005. We modified their questionnaire, using questions only relevant to the purposes of our study.

The objectives of our questionnaire were twofold: to describe the structure, demographics and characteristics of the member practices, and to compare the results to the national profile of Irish general practice [5]. Data requested with regard to patients included total practice list size, General Medical Services (GMS) list size, and breakdown of patient numbers according to age and sex categories. Ireland has a two-tier health system. General Medical Services (GMS) eligible patients are those to whom free primary care and medications are available in Ireland and account for 29% of the population [6]. The remainder, referred to as 'private patients', whose income is above a certain level, are responsible for their own primary health care costs. GMS eligible patients therefore broadly represent the least affluent sector of the community.

Instructions on how to extract required data from various general practice computer software packages were enclosed with the above questionnaire. One postal and one email reminder were subsequently sent.

A dedicated WestREN website was also established and linked to the NUI Galway website (http://westren.nuigalway.ie).

Ethical approval was granted by the Chair of NUI Galway Research Ethics Committee.

## Results

Of the 88 practices in the general practice tutor database, 71 were recruited to the network. Responses were received from 52 practices after 2 reminders. The following statistics refer to those practices that responded to the questionnaire.

All practices were combined GMS and private. The estimated total (GMS plus private) number of patients involved was 224,504, with a mean total list size of 4,776 (SD 3,747, Standard Error 547). The mean GMS list size was 1,358 patients (SD 851, Standard Error 120). The mean percentage of male and female patients per practice was 48.3% (SD 5.4, Standard Error 0.8) and 51.2% (SD 5.5, Standard Error 0.9) respectively.

Half of all practices were in a rural location, with one quarter located in an urban setting and another quarter in a mixed location. Ninety-four per cent of general practitioners practiced from purpose-built and adapted premises, with 5.7% having premises attached to their residence.

Ninety-six per cent of general practitioners used appointment systems with 57.6% using appointment only. A small minority (3.8%) of practices operated on a 'walk-in only' basis.

All practices surveyed were computerised, with 81% describing their practices as 'fully computerised' (defined by the authors in the questionnaire as using a computer for all consultation notes, prescriptions and patient-related correspondence). Of the 19% who were not using computerisation to its full extent, all used computers to generate repeat prescriptions, 60% recorded consultation notes on computer, 80% made use of computers for recording outgoing correspondence and one third used them to scan incoming correspondence electronically. The most popular software packages in use were Health One (38.4%), followed by GP Dynamic (23%), Socrates (17.3%), GP Mac (9.6%) and Helix Practice Manager (7.7%).

Most (59.6%) of the responding general practitioners were coding chronic diagnoses with 20% of responders coding individual consultations.

Twenty-five per cent of general practitioners were operating single-handedly with the majority of practices having at least two general practitioners working in the practice (time commitment not specified), and a mean number of general practitioners of 2.4 (SD 1.3, Standard Error 0.2). Ninety-two per cent of practices employed a practice nurse with 30% employing more than one nurse and the mean number of nurses employed being 1.3 (SD 0.6, Standard Error 0.09).

The majority (61%) of practices used a co-op system to cover their out of hours commitment, with just under a fifth using an external rota system, 3.8% of practices relying on an internal rota system, and 13% using a combination of rota and co-op, e.g. the former during weeknights and the latter at weekends.

Forty-two per cent of practices had been involved in the local general practice training scheme and 60% in general practice oriented research in the preceding three years.

### Relationship to national profile

These results were compared to those of O'Dowd et al. [5] in their national surveys of general practitioners in 1992 and 2005 (see Table 1). In 2005 this would have equated to 545 practices countrywide. Compared to the national profile, WestREN practices appear somewhat larger and more likely to be purpose built in rural areas. Trends apparent between 1982 and 1992, such as computerisation and practice nurse availability, appear to be continuing.

**Table 1:** Comparison of National and WestREN profiles

	**O'Dowd et al. 1992 **[5]	**O'Dowd et al. 2005 **[5]	WestREN 2009
No. of Practices	428	545	71
Response Rate	68%	87%	73%
**Practice Type**			
GMS + Private	91%	96%	100%
Private Practice only	9%	4%	0%
**GMS List Size**			
<500	25%*	27%*	4%
1,000-1,999	27%*	21%	38%
>2,000	3%*	<2%	14%
**Practice Location**			
Rural Location	33%	21%	50%
Urban Location	47%	43%	25%
Mixed Location	20%	36%	25%
**Premises**			
Purpose-built premises	27%	43%	54%
Adapted premises	46%	46%	40%
Attached to residence	27%	11%	5.7%
**Practice Organisation**			
Computerisation	27%	89%	100%
Appointment system	58%	81%	96%
**Out of Hours**			
Internal Rota	25%*	5%*	4%
External Rota	60%	15%*	19%
Co-op	0%	42%	61%
**Practice Staff**			
Single-handed GP practice	57%*	35%	25%
Practice nurse (FT and PT)	17%*	75%	92%
**Education**			
Involved in postgraduate training	8%	18%	42%

*These figures were obtained by extrapolation from graph in O'Dowd et al. [5]

## Discussion

We have described the characteristics of a university-affiliated general practice research network in Ireland. At time of writing, this research network comprises 71 general practices and 224,504 patients. Compared to other international research networks, for example in England where one well-recognised country-wide primary care research network exists comprising central coordination of eight distinct local networks [4], WestREN is clearly not as well-established. However, it is still in the early stages of maturation and continues to recruit new members.

In O'Dowd et al.'s study [5], the authors made comparisons between similar work in 1982, 1992 and 2005. As found in our survey, O'Dowd et al. [5] noted a decrease in the number of general practitioners in private practice alone, from 11% in 1982 to 4% in 2005. Although the O'Dowd et al. study did not give precise figures in relation to estimated practice list sizes, both studies demonstrated a growth in general practitioners with GMS list sizes from 1,000-1,999 and >2,000 patients. Our study demonstrated a fall in the number of practices with GMS lists <500 patients. In addition to this we have also illustrated a reduction in the number of single-handed general practitioners.

Our results also echo the previously noted trend away from practices attached to residences - this percentage went from 27% in 1992 to 11% in 2005. Our figures reiterated the previously reported increase in use of appointment systems and the move away from 'walk-in' surgeries: only 2 practices used a 'walk-in only' system.

There is a definite move towards maximisation of the use of information technology, with 100% of our practices describing themselves as computerised, and 81% of these being fully computerised (i.e. using computers for consultation notes, repeat scripts, incoming and outgoing correspondence). Although only a fifth of practices were coding individual diagnoses (much of which is software package dependant), 60% of our practices were coding chronic diagnoses.

Sixty per cent have been involved in general practice oriented research to date. Thus it is not surprising that such practices have opted to participate in a research network. Both of the above facts will certainly prove helpful in the making the best of the capabilities of the research network, and give an indication as to the involved practices' readiness for research.

### Limitations

There are inherent challenges in accurate list size estimation in Ireland, particularly in relation to private patient numbers. Whilst a 'panel list' is available for all GMS eligible patients, practices do not have complete registers of regularly attending private patients. In addition to this, there is no universal registration system; therefore it is possible that private patients can be registered with several different practices. Also, some patients may have registered with a practice for a one-off visit, and may not have presented again following this. With this in mind, in our study practices were asked to count private patients seen in the last three years only. Even still, accurate estimation of these figures and age/sex breakdown was limited in some cases due to difficulty extracting this data easily from certain general practice software programmes.

This factor was also mentioned in the O'Dowd et al. study [5] and it was pointed out that many of the private patient list figures were estimates on the part of the practitioner involved.

There were also geographical limitations to our study, as it included 71 practices from the West of Ireland only. Although WestREN practices are larger and more likely to be rural, they are broadly representative of Irish general practice as a whole.

### Future plans

From an organisational perspective, previous research networks have been described as having different approaches to leadership: 'top down' (where there are strong institutional links and research projects are led by experienced researchers), 'bottom up' (where practitioners develop their own ideas and the network is led by a peer group) or 'whole system leadership' (where there is a multidisciplinary executive and coalitions of interested people including novices and experienced researchers are developed) [2]. We envisage WestREN having a whole system approach to leadership. We are encouraging member practices to generate their own research ideas, as well as becoming involved in university-led research initiatives.

It is planned to conduct further research through WestREN practices in the near future. To this end we have obtained funding for research bursaries in an effort to encourage and assist individual practices to develop research ideas of their own. Relevance or applicability of the results of this research depends on its generalisability, i.e. whether these results can be realistically applied to a particular group of patients in routine primary care practice [7]. We feel that the practices in WestREN are broadly representative of the national profile and thus would have the potential to produce research results that are generalisable.

WestREN continues to recruit new members and is expanding beyond its strict geographical location.

## Conclusions

WestREN is an academic general practice research network in Ireland. Survey of its initial membership confirms practices are broadly representative of the national profile and has provided us with valuable information on the current and changing structure of Irish general practice. We hope that the network itself will be a rich resource for both research and education.

Further details can be obtained by visiting the WestREN website at http://westren.nuigalway.ie

## Competing interests

The Discipline of General Practice, NUI Galway has received unrestricted educational funding from MSD, Menarini and Pfizer pharmaceutical companies. This funding has been solely used to support educational meetings for general practitioners who take medical students from NUI Galway.

## Authors' contributions

LGG and AWM participated in original conception of the research network, participated in study design and supervised data analysis. KK participated in design of the study, distributed questionnaires, collected and analysed data, drafted the original manuscript and is corresponding author. NOB participated in design of the study and questionnaire distribution. AV designed the research network website. All authors critically reviewed the manuscript and gave their final approval of the version of the manuscript submitted for publication.

## Pre-publication history

The pre-publication history for this paper can be accessed here:

http://www.biomedcentral.com/1471-2296/11/74/prepub

## Supplementary Material

Additional file 1 **Study Questionnaire**. This is a copy of the study questionnaire which was distributed to all WestREN member practices for the purposes of this study. Click here for file
