# Supplementary material for: WestREN: a description of an Irish academic general practice research network
Source: BMC Fam Pract. 2010 Oct 6;11:74. doi: 10.1186/1471-2296-11-74 (PMC2958962; doi:10.1186/1471-2296-11-74)
Supplement: Additional file 1 — Study Questionnaire. This is a copy of the study questionnaire which was distributed to all WestREN member practices for the purposes of this study. [file 1471-2296-11-74-S1.PDF]

Questionnaire – WestREN Confidential Practice Questionnaire

Section A. Practice Structure & Characteristics

1. **Practice Type:** (please circle one): Private Only GMS & Private

2. **Please estimate size of practice (Please refer to the attached guidance sheet for directions on how to compile the following figures on the relevant software system)**

Total List Size: ______
GMS list size: ______
Males (Number or % or both): __________
Females (Number or % or both): __________

Age breakdown (Number or % or both):

0-9 _____	40-49______
10-19 _____	50-59______
20-29 _____	60-69______
30-39 _____	70+ ______

3. **Practice Location:** (please circle one)

Rural Urban Mixed rural-urban

4. **Type of main practice premises:** (please circle one)

Purpose-built Adapted Attached to residence

5. **Appointment systems:** (please circle one)

Appointment only Walk-in only Mixed Appointment/Walk-in

Section B. Computerisation

6. **Are computers used in your practice** (please circle one)? Yes No

7. **If so to what extent are computers used** (please circle as appropriate)?

-**Full** : For all consultation notes, prescriptions and patient correspondence

OR

-**Partial** (please circle all appropriate options)

Repeat prescriptions
Consultation notes
Outgoing referral letters/correspondence
Scanning incoming correspondence

CONTINUED OVERLEAF……

8. Practice Software Package: (please circle one)

Socrates / GP Dynamic / GP Mac / Helix Practice Manager / Health One / Other (Please specify)_______

9. Do you code chronic diagnoses for each patient (eg diabetes/asthma)?
Yes No

10. Do you code each consultation diagnoses for each patient (eg UTI)?
Yes No

11.If so which coding system do you use? (please circle one or both)

ICPC ICD 10

Section C: Practice Staff

12. Number of GPs active within practice (excluding GP trainees) _______

13. Does the practice have a nurse? (Please circle) Yes No

14. If yes how many nurses?________

Section D: Out of Hours Duty

15. When you are 'Off Duty' (nights,weekends, public hols etc) **which of the following deputising arrangements do you employ?** (Please circle one)

Co-op / Internal Practice Rota System / Rota System involving doctors outside your practice / Deputising service / Locum/ Other________________

Section E: Educational activity of practice

16. In the past three years has this practice been a training practice for the local GP training scheme?(Please circle one) Yes No

17.In the past three years has the practice been involved in any general practice oriented research?(Please circle one) Yes No
